# Multiparametric Mechanistic Profiling of Inotropic Drugs in Adult Human Primary Cardiomyocytes

**DOI:** 10.1038/s41598-020-64657-2

**Published:** 2020-05-06

**Authors:** Najah Abi-Gerges, Tim Indersmitten, Ky Truong, William Nguyen, Phachareeya Ratchada, Nathalie Nguyen, Guy Page, Paul E. Miller, Andre Ghetti

**Affiliations:** 1 AnaBios Corporation, San Diego, CA 92109 USA; 2 Present Address: Arena Pharmaceuticals, San Diego, CA 92121 USA

**Keywords:** Chemical biology, Drug discovery, Cardiology

## Abstract

Effects of non-cardiac drugs on cardiac contractility can lead to serious adverse events. Furthermore, programs aimed at treating heart failure have had limited success and this therapeutic area remains a major unmet medical need. The challenges in assessing drug effect on cardiac contractility point to the fundamental translational value of the current preclinical models. Therefore, we sought to develop an adult human primary cardiomyocyte contractility model that has the potential to provide a predictive preclinical approach for simultaneously predicting drug-induced inotropic effect (sarcomere shortening) and generating multi-parameter data to profile different mechanisms of action based on cluster analysis of a set of 12 contractility parameters. We report that 17 positive and 9 negative inotropes covering diverse mechanisms of action exerted concentration-dependent increases and decreases in sarcomere shortening, respectively. Interestingly, the multiparametric readout allowed for the differentiation of inotropes operating via distinct mechanisms. Hierarchical clustering of contractility transient parameters, coupled with principal component analysis, enabled the classification of subsets of both positive as well as negative inotropes, in a mechanism-related mode. Thus, human cardiomyocyte contractility model could accurately facilitate informed mechanistic-based decision making, risk management and discovery of molecules with the most desirable pharmacological profile for the correction of heart failure.

## Introduction

Myocardial contractility (inotropy) is an essential property of cardiac function and must be maintained at constant physiological level. Non-cardiac drugs causing unintended contractility effects can lead to adverse cardiac events including contractile dysfunction and heart failure, limiting the utility of novel innovative treatments^1–3^. Moreover, the traditional models, animal-based and iPSC-derived cardiomyocytes, currently in use to assess changes in contractility, do not fully capture the physiology of human primary cardiomyocytes^4,5^ and lack mature inotropic mechanisms^6–10^. These major shortcomings render the existing models inadequate when trying to reliably identify toxicity risks at the preclinical stages of drug discovery. Similarly, the current animal- or iPSC-based models are not reliable when contractility is a critical endpoint for establishing a molecule’s potential to treat heart failure; patients with reduced ejection fraction continue to have an unfavourable prognosis with high morbidity and mortality^11^. Consequently, heart failure remains a major unmet medical need with a prevalence that continues to rise^12^. From a therapy development standpoint, some key challenges originate from the incomplete understanding of the underlying pathophysiological mechanisms^13^ and the lack of a relevant model^14,15^ to aid the selection, at the preclinical stage, of the best heart failure drug candidates for clinical development. The inability of the current animal models to recapitulate all critical elements of the physiological, pharmacological and pathological states has resulted in limited translation and high clinical attrition^15,16^. Furthermore, recent iPSC-derived cardiomyocyte models can only help to clarify therapies that may be beneficial in treating inherited forms of heart failure, but not acquired forms that are often the result of abnormal physiological insults^17^. It would be highly valuable to develop an adult human primary cardiomyocyte contractility model to facilitate both the early identification of cardiotoxicity risks as well as of new drugs with the pharmacological profile for the correction of contractility deficit. Such a human-relevant contractility platform could enable the generation of reliable and predictive data with a higher rate of successful clinical translation.

Historically, adult human cardiac tissues and cardiomyocytes have been generally unavailable for preclinical studies, except for the occasional use of surgical discards of atrial appendages and ventricular walls^7,18–24^. The use of primary human cardiomyocytes in drug discovery as well as in basic research has been hindered by major challenges related to the limited availability of human donor hearts, the inconsistent quality and the limited yield of isolated cells. Our laboratory has focused on the development of strategies and tools to bridge the translational gap by enabling large scale utilization of human primary cells and tissues. Access to cardiac tissues and cardiomyocytes from healthy as well as heart failure hearts, obtained from organ donors, allows for physiological-, biochemical- and omics-based investigation of the pathophysiology to a level unattainable in the past^9,25^. In addition, we have developed functional assays aimed at characterizing drug activity using physiological end points, both in isolated trabeculae tissue sections with action potential recordings^25–27^ as well as isolated ventricular myocytes with contractility measurements^9^. We now report on the utilization of adult human primary cardiomyocytes in a contractility assay and the testing of inotropic drugs. We have used human cardiomyocytes from ethically consented organ donors to measure contractility transients using a validated bright-filed imaging-based platform^9^. We relied on changes in contractility parameters to infer both drug-induced inotropic effect (maximum amplitude of contraction expressed as sarcomere shortening) as well as the mechanisms of action based on clustering and principal component analyses of a set of 12 contractility parameters. The relevance of this approach was addressed using a panel of 26 inotropes (17 positive, 9 negative) spanning a variety of mechanisms.

## Results

### Effects of inotropes on maximum amplitude of contraction in adult human primary cardiomyocytes

**Figure 1:**
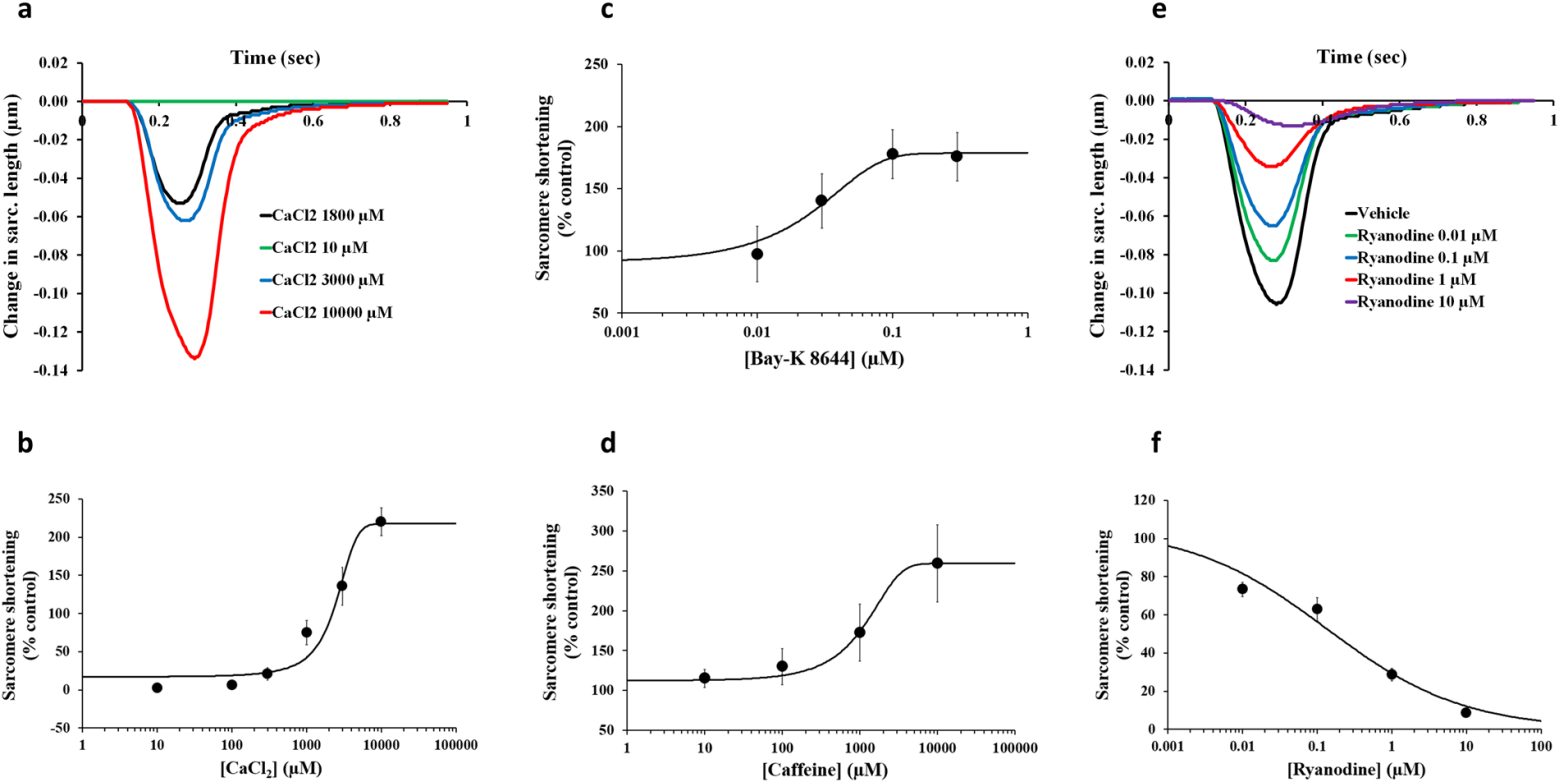
Effects of CaCl_2_, BayK-8644, Caffeine and Ryanodine on human cardiomyocyte contractility. **(a)** Typical contractility transients recorded from an adult human primary ventricular myocyte in the presence of vehicle control and after exposure to CaCl_2_ at 10, 1800, 3000 and 10000 µM**. (b)**, **(c)** and **(d)** C-E curves generated with CaCl_2_, BayK-8644 and Caffeine, respectively. **(e)** Typical contractility transients recorded from an adult human primary ventricular myocyte in the presence of vehicle control and after exposure to Ryanodine at 0.01, 0.1, 1 and 10 µM**. (f)** C-E curve generated with Ryanodine. Each C-E curve plot shows the effect of a test article on sarcomere shortening as well as the mean data points and the fitted C-E curve. IonWizard software (v1.2.22, www.ionoptix.com) and SigmaPlot v14.0 (www.systatsoftware.com) were used to generate the representative contractility transients and fitted C-E curves, respectively.

**Figure 2:**
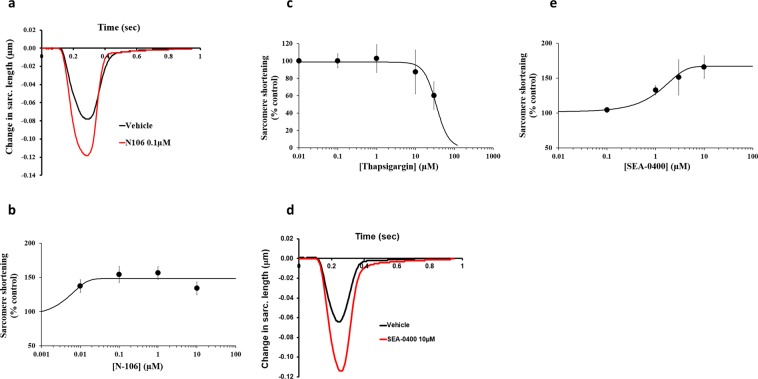
Effects of N-106, Thapsigargin and SEA-0400 on human cardiomyocyte contractility. **(a)** Typical contractility transients recorded from an adult human primary ventricular myocyte in the presence of vehicle control and after exposure to N106 at 0.1 µM**. (b,c)** C-E curves generated with N-106 and Thapsigargin, respectively. **(d)** Typical contractility transients recorded from an adult human primary ventricular myocyte in the presence of vehicle control and after exposure to SEA-0400 at 10 µM**. (e)** C-E curve generated with SEA-0400. Each C-E curve plot shows the effect of a test article on sarcomere shortening as well as the mean data points and the fitted C-E curve. IonWizard software (v1.2.22, www.ionoptix.com) and SigmaPlot v14.0 (www.systatsoftware.com) were used to generate the representative contractility transients and fitted C-E curves, respectively.

**Figure 3:**
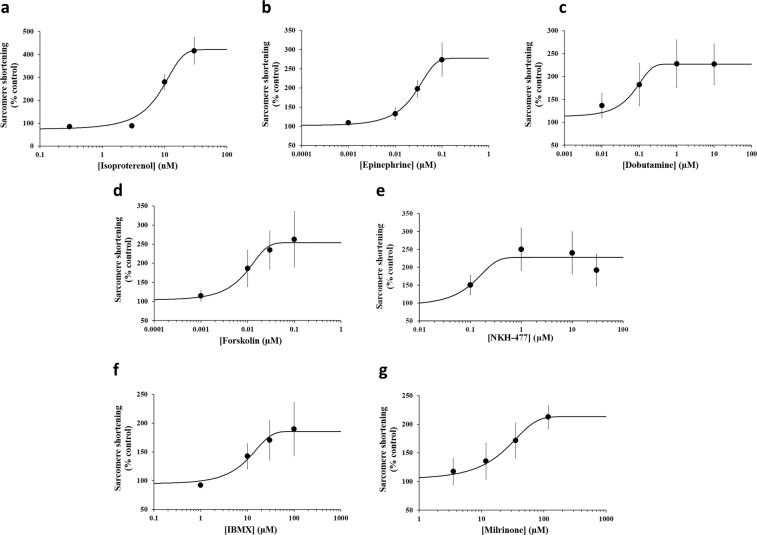
Potency information generated with positive inotropes from human cardiomyocytes paced at 1 Hz pacing frequency. Typical cumulative C-E curves generated by human cardiomyocyte contractility measurements with 7 positive inotropic drugs: Isoproterenol **(a)**, Epinephrine **(b)**, Dobutamine **(c)**, Forskolin **(d)**, NKH-477 **(e)**, IBMX **(f)** and Milrinone **(g)**. Each plot shows the effect of a test article on sarcomere shortening as well as the mean data points and the fitted C-E curves. SigmaPlot v14.0 (www.systatsoftware.com) was used to generate the fitted C-E curves.

**Figure 4:**
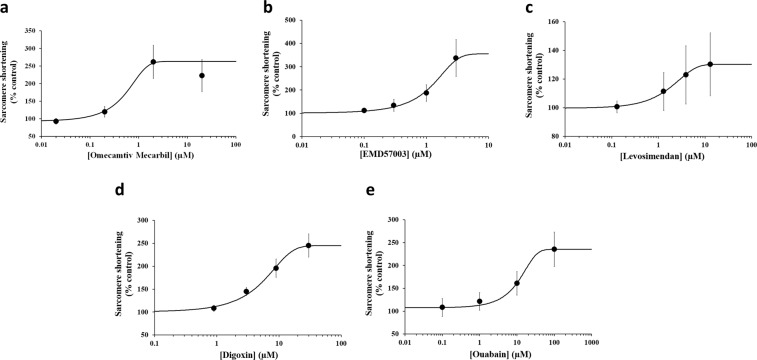
Potency information generated with positive inotropes from human cardiomyocytes paced at 1 Hz pacing frequency. Typical cumulative C-E curves generated by human cardiomyocyte contractility measurements with 5 positive inotropic drugs: Omecamtiv Mecarbil **(a)**, EMD57003 **(b)**, Levosimendan **(c)**, Digoxin **(d)** and Ouabain **(e)**. Each plot shows the effect of a test article on sarcomere shortening as well as the mean data points and the fitted C-E curves. SigmaPlot v14.0 (www.systatsoftware.com) was used to generate the fitted C-E curves.

We first addressed the role of systolic Ca^2+^, its uptake and removal in the contraction of adult human primary cardiomyocytes from ethically consented organ donors. The data show that sarcolemmal Ca^2+^ influx, via the voltage-gated Ca^2+^ channel, and sarcoplasmic reticulum (SR) Ca^2+^ release, via the ryanodine receptor, regulated the systolic Ca^2+^. In order to reveal this dependence, we measured the effects of various pharmacological manipulations on sarcomere shortening: first, by varying the level of extracellular Ca^2^ (Fig. 1a,b), activating the Ca^2+^ channel with Bay-K 8644 (Fig. 1c), then activating or inhibiting the ryanodine Ca^2+^ release channel receptor with Caffeine (Fig. 1d) or Ryanodine (Fig. 1e,f), respectively. While low μM concentration of Ca^2+^ led to full inhibition of sarcomere shortening, cumulatively increasing the Ca^2+^ concentration augmented the contraction and allowed to construct a Ca^2+^ concentration-response curve with an EC_50_, the molar concentration producing 50% increase, of 2800 μM, (n = 5 cells; Fig. 1b). Additionally, Bay-K 8644-induced activation of the Ca^2+^ channel increased sarcomere shortening with an EC_50_ of 0.04 μM (n = 5 cells; Fig. 1c). Moreover, activation and inhibition of the ryanodine receptor Ca^2+^ release channel with Caffeine and Ryanodine were found to increase (Caffeine EC_50_ 1064 μM, n = 5 cells; Fig. 1d) and decrease sarcomere shortening (Ryanodine IC_50_ = 0.16 μM; n = 4 cells; Fig. 1f). We also found that the activation and inhibition of the sarco/endoplasmic reticulum Ca^2+^ ATPase pump (SERCA) with N106 and Thapsigargin, respectively, influenced the pumping of Ca^2+^ from the cytoplasm into the SR and therefore lead to increasing (EC_50_ = 0.006 μM; n = 5 cells; Fig. 2a,b) and decreasing (IC_50_ > 30 μM; n = 4 cells; Fig. 2c) sarcomere shortening, respectively. We also confirmed the ability of the Na^+^/Ca^2+^ exchanger (NCX) to influence the cytoplasmic Ca^2+^ concentration with the testing of SEA-0400, an NCX inhibitor. SEA-0400 was also found to induce an increase in sarcomere shortening (EC_50_ = 1.24 μM; n = 5 cells; Fig. 2d,e). A concentration-dependent increase in sarcomere shortening was also observed with *β*-adrenergic agonists (Isoproterenol, EC_50_ = 9.3 nM, n = 4 cells; Epinephrine, EC_50_ = 0.03 μM, n = 4 cells; Dobutamine, EC_50_ = 0.07 μM, n = 5 cells), adenylyl cyclase activators (Forskolin, EC_50_ = 0.01 μM, n = 5 cells; NKH-477, EC_50_ = 0.09 μM, n = 6 cells) and phosphodiesterase (PDE) inhibitors (IBMX, a non-selective PDE inhibitor, EC_50_ = 11.9 μM, n = 5 cells; Milrinone, a PDE type 3 inhibitor, EC_50_ = 23.5 μM, n = 4 cells) (Fig. 3; Supplementary Fig. 1). Compared to this, increase of sarcomere shortening with cardiac myosin activators (Omecamtiv Mecarbil, EC_50_ = 0.6 μM, n = 5 cells; EMD-57003, EC_50_ = 1.2 μM, n = 5 cells), a Ca^2+^ sensitizer (Levosimendan, EC_50_ = 1.9 μM, n = 4 cells) and Na^+^/K^+^ ATPase inhibitors (Digoxin, EC_50_ = 6.1 μM, n = 7 cells; Ouabain, EC_50_ = 11.2 μM, n = 5 cells) was also concentration-dependent, though the increase in sarcomere shortening was less marked with Levosimendan (Fig. 4; Supplementary Fig. 2). Taken together, these data demonstrate: (1) the full functionality of the excitation-contraction coupling in isolated adult human primary cardiomyocytes, (2) isolated human cardiomyocytes exhibit concentration-effect relationships consistent with the known pharmacology of the tested inotropes and (3) these cells are able to engage different inotropic mechanisms of action that are known to affect human cardiac contractility.

Next, we assessed sarcomere shortening intra-heart variability in response to 0.03 µM Isoproterenol (9 different human hearts) or 10 µM Verapamil (5 different human hearts) (Supplementary Fig. 3, Supplementary Table 1). We found no statistically significant difference across cells from different hearts, in the positive inotropic effects of Isoproterenol (Supplementary Fig. 3a) and negative inotropic effects of Verapamil (Supplementary Fig. 3b).

### Mechanistic differentiation of inotropes

**Figure 5:**
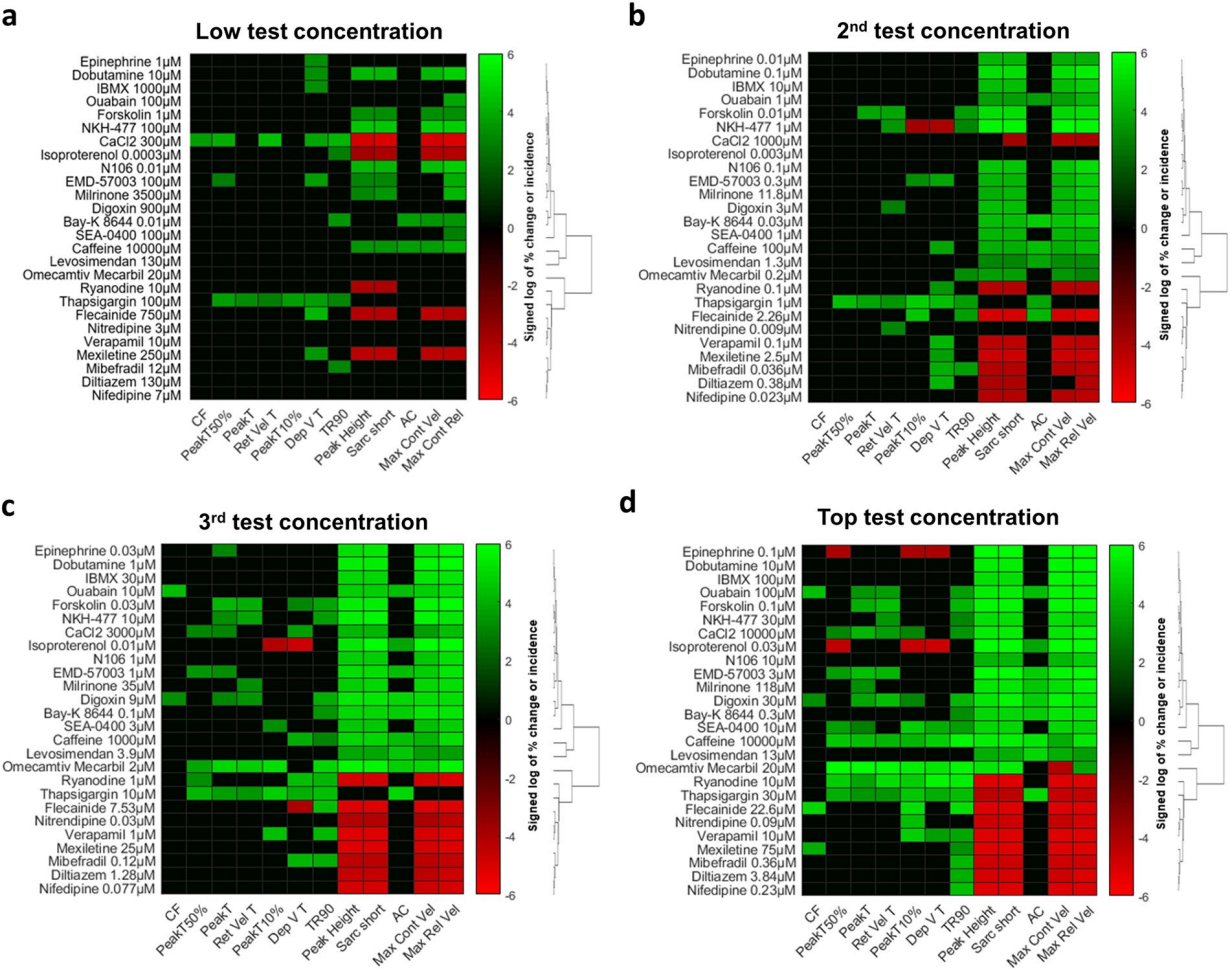
Classification of subsets of both positives as well as negative inotropes, in a mechanism-related mode after exposure to low **(a)**, 2^nd^ **(b)**, 3^rd^ **(c)** and top **(d)** test concentrations. Right-skewed percentage data of incidence (AC and CF) and change (all other contractility parameters) were normalized with a signed log transformation. Heatmap representations were created with a cluster analysis using dendrograms and partitions given the elbow criterion. Red and green colors indicate decrease and increase of >25% and >10% change, respectively. **Black** color indicate no effect (<−25% <% change <10%).

The reliance on sarcomere shortening allows for the detection of drug-induced inotropic activity, which is tightly coupled to intracellular Ca^2+^ regulation. To investigate if the measurement of contractility transients can distinguish separate mechanisms of inotropic drugs, we classified inotropes based on the drug-induced changes in a set of 12 parameters (Fig. 5; Supplementary Fig. 4). We applied this approach to a panel of 26 inotropes, covering fourteen different mechanisms (Fig. 5; Supplementary Table 2). To guide the identification of the minimal set of parameters that are associated with the distinct mechanisms, we classified inotropes and contractility measures into hierarchical clusters using the clustering function (hclustfun) in R Core Team (2018). Inotropic drugs and contractility measures at all concentrations were sorted based on the observed changes at the highest concentration tested. A heat map representation of the hierarchical clusters of drug-induced changes can be seen in Fig. 5. We found that the parameters describing the kinetics of contractility transient were associated with positive and negative inotropes (Figs. 1,2 and 5; Supplementary Figs. 1 and 2). Based on their kinetic profile, distinct subsets of both positives as well as negative inotropes could be classified according to their mechanisms of action. As can be seen from the heat maps, drug-induced changes to the kinetics of the contractility transient were concentration-dependent with the clusters becoming more distinct from each other as drug concentration increased. Next to kinetics, contraction failure (CF) and aftercontraction (AC) were indicators for distinct mode of action. For example, although inhibitors of voltage-gated Na^+^ channels (Flecainide and Mexiletine) and voltage-gated Ca^2+^ channels (Diltiazem, Mibefradil, Nifedipine, Nitrendipine and Verapamil) were both found to decrease cardiomyocyte contractility, the multiparametric readout allowed to differentiate Na^+^ channel-from Ca^2+^ channel- antagonists; only Na^+^ channel inhibitors, but not Ca^2+^ channel-inhibiting drugs, were found to induce CF (Fig. 5d). This finding correlated with the role of Na^+^ and Ca^2+^ channels in excitation-contraction coupling. The differentiation of drugs inhibiting ryanodine receptor Ca^2+^ release channel and SERCA pump provides another example highlighting the ability of the multiparametric readout to classify drugs operating via distinct mechanisms. Although modulation of both targets inhibited sarcomere shortening and resulted in similar effects on 10 contractility parameters, the effects on AC allowed differentiating a ryanodine receptor Ca^2+^ release channel inhibitor from an inhibitor of the SERCA pump. Only Thapsigargin, but not Ryanodine, induced AC (Fig. 5d). This finding is consistent with the role of ryanodine receptor Ca^2+^ release channel and SERCA pump in excitation-contraction coupling. Both Isoproterenol and Omecamtiv Mecarbil caused positive inotropic effect by increasing sarcomere shortening, but lusitropic activity (i.e., relaxation rate of the cardiomyocyte contractility) allowed to differentiate the two drugs. While Isoproterenol promoted positive lusitropy, enabling the cardiomyocyte to relax more rapidly, Omecamtiv Mecarbil was associated with negative lusitropy, allowing the cardiomyocyte to relax less rapidly (Fig. 5d). These findings are consistent with the effects that follow activation of the β-adrenoceptor pathway and cardiac myosin.

**Figure 6:**
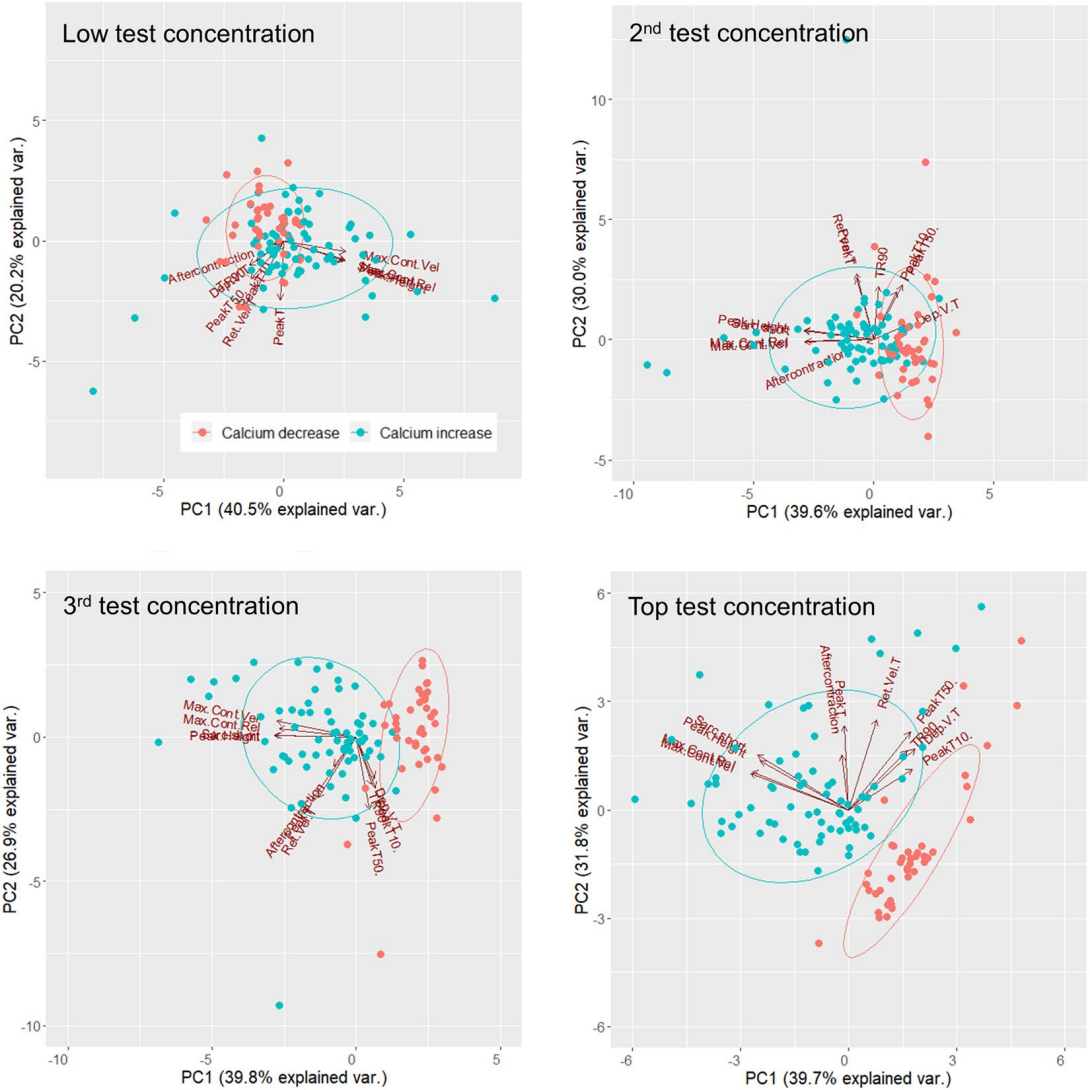
Segregation of inotropes based on the ability to increase or decrease intracellular Ca^2+^. Aqua and red cluster borders mark the 75% confidence interval for predicting if a novel unknown compound induces a positive or negative inotropic effect via increasing or decreasing intracellular Ca^2+^, respectively.

To assess if the Ca^2+^-dependent mechanism of action of inotropes could be predicted with the multiparametric readout, we performed a Principal Component Analysis (PCA) to reduce the dimensionality of all 12 contractility measures, using the function prcomp in R (R Core Team, 2018). Contractility measures were normalized to baseline and the dataset on which the PCA was performed at the highest test concentration consisted of 40 (red color) and 70 (blue color) cells from 9 negative and 14 positive inotropes, respectively (Fig. 6). Normality of data was confirmed by plotting normal histograms using the function qqnorm (data not shown). Ellipses indicate confidence intervals of 0.75. Scree plots (Supplementary Fig. 5) and separation of ellipses showed that at the 3^rd^ highest or top test concentrations, 3 principal components were sufficient to predict if a drug increased or decreased intracellular Ca^2+^ (Fig. 6). In contrast, at the lowest and 2^nd^ lowest concentrations tested, there was substantial overlap of ellipses (Fig. 6). At the top test concentration, the cumulative proportion of explained variance of the first three principal components was 0.794. The percentage of variances that was explained by the first three principal components were 39.74 ± 2.09 (% ± std) for PC1, 31.76 ± 1.87 for PC2 and 7.90 ± 0.93 for PC3. The directionality of vectors showed that changes in sarcomere shortening, Peak Height, Max Cont Vel and Max Rel Vel were most predictive for changes in intracellular Ca^2+^. These findings suggest that the Ca^2+^-dependent mechanism of action of a novel drug can be predicted, provided that the drug concentration can change the directionality of vectors. Thus, both hierarchical clustering of contractility parameters and PCA yield insights into the mechanism of action of both positive as well as negative inotropy.

## Discussion

Over the last few years, new methods and strategies have emerged that could bridge the translational gap in cardiac risk assessment and cardiac disease drug discovery^28,29^. One rapidly developing new area leverages the large-scale utilization of human primary cells and tissues, obtained from organ donor hearts^9,25–27^. These studies functionally characterize drug activity using physiological endpoints measured in *ex vivo* human trabeculae to address pro-arrhythmia risk^25–27^ as well as in isolated adult human ventricular cardiomyocytes to simultaneously predict risks associated with negative inotropic activity and pro-arrhythmia^9^. In an extension of the previous work, the adult human primary cardiomyocyte contractility assay described in the present study has the potential to simultaneously predict drug-induced positive inotropic activity and identify specific inotropic mechanism of action.

When the drug-induced changes in contractility were analysed using a multiparametric readout, we were able to differentiate drugs based on their distinct mechanisms of action. Hierarchical clustering of contractility parameters, coupled with principal component analysis, enabled the classification of subsets of both positives as well as negative inotropes, in a mechanism-related mode. For example, the parameter CF allowed to differentiate Na^+^ from Ca^2+^ channel inhibitors. Following inhibition of Na^+^ channel, the action potential is not initiated, excitation-contraction coupling is inhibited, and consequently the cardiomyocyte fails to contract. On the contrary, the inhibition of Ca^2+^ channel decreases the sarcolemmal Ca^2+^ influx without affecting the rising of the action potential and therefore the cardiomyocyte contracts. Moreover, the parameter AC permitted to distinguish ryanodine receptor Ca^2+^ release channel from SERCA pump inhibitors. Following the inhibition of the SERCA pump, the Ca^2+^ pumping activity of the Ca^2+^ ions from the cytoplasm into the sarco/endoplasmic reticulum is diminished, cytosolic Ca^2+^ level is increased, and subsequently ACs are induced. Quite the opposite, the inhibition of ryanodine receptor Ca^2+^ release channels inhibits the release of Ca^2+^, cytosolic Ca^2+^ level is decreased, and ACs do not occur. The multiparametric readout allowed as well to differentiate between drugs possessing positive or negative lusitropic activities from those with no effect on the relaxation rate of the cardiomyocyte contractility. Thus, this novel approach will enable the identification of the inotropic potential of novel molecules at the preclinical stages of drug development and facilitate informed mechanistic-based decision-making for more effective management of contractility risk. Alternatively, in the context of heart failure drug discovery, the approach described here could enable the selection of drugs with the most desirable pharmacological profile for the correction of specific forms of contractility deficit.

Cardiomyocyte excitation-contraction coupling is central to achieving and maintaining physiological myocardial contractility. Drugs that decrease or increase cardiac contractility may have safety consequences and may be associated with risk of decreased left ventricular ejection fraction^1,2,9,30^ or increased arrhythmic mortality in patients with preexisting cardiac diseases^31–33^. Despite the potentially unlimited availability, animal-derived primary cardiomyocytes and iPSC-derived cardiomyocytes currently used for preclinical safety studies have physiological and pharmacological shortcomings that limit their utility in the context of inotropy-related risk assessment^4–10,15,34–42^. Similarly, these models lack the ability to identify drugs with a desirable positive inotropic potential for the correction of heart failure^43–49^. For example, the shortcomings include irregular cell shape morphology^50,51^, disorganized sarcomeric structure^52^, deficient t-tubule structure^53,54^, non-uniform distribution of ryanodine receptor Ca^2+^ channel release^50,55^, no predominance of PDE type 3 and no contribution of *β*2-adrenoceptors to the *β*-adrenergic positive inotropic effect^7^, low expression of Ca^2+^-handling proteins^7,38,56^, low sarco/endoplasmic reticulum Ca^2+^ store^56^, leaky sarco/endoplasmic reticulum without a mature terminating mechanism^57^, low expression of ion channels^10,58–64^, lack of cardiac chamber specificity^65^, negative force-frequency relationship^54,66^. Consequently, data derived from these models must be interpreted with caution and adult human primary cardiomyocytes provide a more relevant platform for enhancing our knowledge of human ventricular physiology and performing drug activity profiling at the preclinical stage. Over the last few decades, adult human cardiac tissues and cardiomyocytes, primarily from explanted hearts of heart transplant recipients, have been used to study cardiomyocytes and modulation of their contractile function while profiling the same few inotropic agents using different experimental conditions^43,45,47,67–93^. However, the availability of explanted hearts as well as organ donor hearts has been very limited, and this has precluded the routine use of human primary cardiomyocytes in drug discovery.

During the last few years, our laboratory has developed the capability to procure viable human hearts from organ donors, which has enabled large scale utilization of human cardiomyocytes and tissues which are utilized in functional assays aimed at characterizing drug activity using physiologically relevant endpoints^9,25–27^. Our previous studies demonstrated the low donor-to-donor variability with regards to physiological and pharmacological properties of the adult isolated human primary cardiomyocytes^9^ and ventricular trabeculae^25^. In addition, these *ex-vivo* models proved to be highly accurate in differentiating cardiotoxic and non-cardiotoxic drugs^9,25,27^. We now further report that cardiomyocytes obtained from multiple donor hearts exhibited consistent and isolation-independent sarcomere shortening in response to two prototypical inotropic agents, Isoproterenol and Verapamil. The data from the current feasibility study also show that the human primary cardiomyocyte contractility model can detect both negative and positive inotropic effects. Moreover, this is the first study reporting the use of a large, diverse panel of established and novel (like Omecamtiv Mecarbil and N106) positive inotropic drugs, with 14 different mechanisms of action, in combination with a physiologically functional adult human primary cardiomyocyte contractility model assessing and reliably predicting positive inotropy. This finding not only confirmed the full functionality of the excitation-contraction coupling in adult human primary cardiomyocytes isolated from organ donor hearts, but also correlated with the clinical effects of the positive inotropes that are currently being used to treat heart failure patients^33,94,95^, undergoing clinical trials^96–100^ or in preparation for Investigation New Drug application^101^. Hence, the ability of human primary cardiomyocytes to provide reliable and predictive data to support heart failure discovery projects; this could facilitate the discovery of innovative therapies that can be used population-wide (e.g. calcitropes, myotropes and mitotropes) by directly modulating the contractile function of cardiomyocytes^33^. However, cardiomyocytes derived from organ donors cannot currently be maintained in culture, which limits their utility in studies aiming at studying gene therapy methods for correcting cardiac diseases. In addition, these cell preparations do not have the regenerative and proliferative potential that has been described in methods that employ stem cells and iPSC-derived cardiomyocytes. This renders the methods described in this paper not applicable to personalized and regenerative therapy. Finally, the panel of drugs and targets that was assessed in this study is not exhaustive and future studies will be needed to evaluate other potential targets, like G-protein-coupled receptors, which modulate numerous signaling pathways in healthy and failing hearts^102^, and cyclase modulators^103^.

Human primary cardiomyocytes (i) allow an integrated evaluation of drug effects on all human cardiac targets, (ii) are predictive of clinical outcomes and (iii) can be used in screening format, we envision a new potential contractility testing paradigm in which the adult human primary cardiomyocyte model can be employed as the first early primary screen for inotropic risk assessment and ranking of drugs. Lead drugs with no or low potential to induce inotropic risk can then be progressed to late preclinical stages of drug development and tested in the human tissue-based contractility model^104^ and *in vivo* regulatory cardiovascular assessment models. Taken together, all these data would provide reliable assessment to guide first-in-human dosing. If data indicate that drugs can be associated with inotropic risk, this undesired liability would first need to be eliminated.

We further found that the human cardiomyocyte model can generate data useful in identifying the possible modes of action. Our study shows that the multiparametric contractility readout allows for the differentiation of drugs operating via distinct mechanisms. Hierarchical clustering analysis, coupled with principal component analysis, enables the classification of subsets of both positives as well as negative inotropes, in a mechanism-related mode. Such mechanistic information can help with the implementation of follow-up screens, including cardiac ion channel assays, to dial out the affinity of drugs to the undesired target.

A similar preclinical strategy can also be proposed to support discovery programs that aim at developing new heart failure treatments. Following the selection of targets, engagement of different chemical series to these targets can be examined in the adult human primary tissue and cardiomyocyte contractility model and cell lines overexpressing the targets, so lead compounds can be selected. During lead optimization and candidate drug selection stages, it is recommended to ensure lead and/or candidate drugs demonstrate ventricular selectivity with no potential to cause pro-arrhythmia. Our proposed strategy provides an opportunity to heart failure projects to test effectiveness and safety of new potential drugs and implement a translational paradigm with no total dependence on extrapolative data from questioned animal studies^15^, and a potential to significantly reduce animal use in research.

In conclusion, the results of the present investigation suggest that adult human primary cardiomyocytes provide a suitable model for the evaluation and detection of inotropic potential of novel drugs, overcoming the limitations of current approaches and providing an integrated physiologically functional model to determine prospective mechanistic insights. This strategy, combined with optimized conditions for storage/distribution and automated large-scale drug testing and analysis, could contribute reducing the contractility risk burden of novel therapeutics and facilitating the identification of molecules with the most desirable pharmacological profile for the treatment of heart failure.

## Methods

### Donor heart procurement

All methods were carried out in accordance with relevant guidelines and regulations. All human hearts used for this study were non-transplantable and ethically obtained by legal consent (first person or next-of-kin) from cadaveric organ donors in the United States. Our recovery protocols and *in vitro* experimentation were pre-approved by IRBs (Institutional Review Boards) at transplant centers within the US OPTN (Organ Procurement Transplant Network). Furthermore, all transfers of the donor hearts are fully traceable and periodically reviewed by US Federal authorities. Donor characteristics, heart number and donor identifier are shown in Table 1 and exclusion criteria were previously described^25^.

**Table 1:** Donor characteristics.

Heart #	Donor identifier	Age	Sex	Ethnicity	BMI	COD	EF (%)
1	171025HHA	33	M	Caucasian	30.3	CVA/ICH	60
2	171116HHA	19	F	Hispanic	25.4	Head trauma	55
3	171216HHA	54	M	African American	25.9	CVA/ICH	50
4	180405HHA	50	M	Caucasian	25.4	Head trauma	70
5	180415HHA	50	M	African American	27.2	Head trauma	65
6	180503HHA	27	M	Hispanic	24.7	CVA/ICH	62
7	181109HHA	59	M	Caucasian	30.8	CVA/ICH	N/A^a^
8	170906HHA^9^	39	F	Caucasian	19.5	AS/Suicide	N/A^a^
9	161115HHA^9^	27	M	Hispanic	25.9	Anoxia	60
10	170822HHA^9^	45	M	Hispanic	24.7	Head trauma	70

F, Female; M, Male; BMI, Body Mass Index; COD, Cause Of Death; EF, Ejection Fraction; CVA, Cerebrovascular Accident; ICH, Intracranial Haemorrhage; AS, Asphyxiation; HH, Human Heart; HHA, HH AnaBios; ^a^Organ procurement organization could not transplant the heart and consequently no echocardiography was performed; N/A, Not available. Nguyen, N. *et al*.^9^.

### Cardiomyocyte contractility measurement

Upon arrival at our laboratory, hearts were re-perfused with ice cold proprietary cardioplegic solution and adult human primary ventricular myocytes were isolated enzymatically from the ventricles^9,25^. Contractility transients were measured as previously described^9,105^. Briefly, cardiomyocytes were placed in a perfusion chamber (FHC Inc., Bowdoin, ME, USA) mounted on the stage of an inverted Motic AE31E microscope (StellarScientific, MD, USA) and continuously perfused from a gravity fed system at 2 ml/min with myocyte Tyrode solution (see composition below) heated to approximately 36 °C using an inline heater (Cell MicroControls, Norfolk, VA, USA). A video-based cell geometry system was used to measure sarcomere dynamics (IonOptix (v7.2.7.138), MA, USA, www.ionoptix.com)^106^. The myocytes were field stimulated at voltage 50% above threshold at a 1 Hz pacing frequency, with a biphasic pulse of 3 ms duration, using a pair of platinum wires placed on opposite sides of the chamber and connected to a MyoPacer EP stimulator (IonOptix). Images were acquired at a rate of 240 Hz using an IonOptix MyoCam-S CCD camera. Digitized images were displayed within the IonWizard acquisition software (IonOptix). Optical intensity data were collected from a user-defined rectangular region of interest placed over the myocyte image. The optical intensity data represent the bright and dark bands corresponding to the Z-bands of the cardiomyocyte. The IonWizard software (v1.2.22) analyzes the periodicity in the optical density along the myocyte detecting the Z-bands by means of a fast Fourier transform algorithm.

The stability of sarcomere shortening transients was assessed by continuous recording for 120 sec in Tyrode’s solution establishing the vehicle control (in 0.1% dimethyl sulfoxide, DMSO). Subsequently, the test article concentration was applied for a minimum of 150 sec period. Four ascending concentrations of the test article were used, providing cumulative concentration-effect (C-E) curves. Analysis was performed using the IonWizard software/Transient Analysis Tool A series of polynomials were fitted to the 5 different phases of the monotonic transient^9^. For each test condition, the values of a set of 10 parameters related to the contractility transient (sarcomere shortening (Sarc. short.), maximum contraction velocity (Max Cont Vel), maximum relaxation velocity (Max Rel Vel), peak height, departure velocity time (Dep V T), return velocity time (Ret Vel T), time to peak (PeakT), time to 10% peak (PeakT10%), time to 50% peak (PeakT50%), time to 90% relaxation (TR90) were calculated from the average of the last 15 contractions and used to quantify test article-induced effects. AC and CF were also used to quantify article-induced effects^9^. An AC was visually identified as change in the slope of the contractility transient that occurred before the next stimulus-induced contraction. CF was also visually identified when the electrical stimulus did not result in a contraction transient. Presence or absence of AC and CF events was determined by examining non-averaged transients for the 150-sec application test article concentration. Results are expressed as mean ± s.e.m. AC and CF were expressed as incidence: number of cells showing events normalized by the total number of cardiomyocytes. Treatment effects on the remaining 10 parameters were expressed relatively to the myocyte’s specific baseline control period. Hill curves were fitted to sarcomere shortening C-E data using SigmaPlot v14.0, (Systat Software Inc., CA, USA, www.systatsoftware.com)^9^ and used to determine EC_50_ (concentration inducing 50% increase in sarcomere shortening).

### Solutions and test articles

The standard myocyte Tyrode solution contained (in mM): NaCl 145, KCl 4, CaCl_2_ 1.8, MgCl_2_ 1, glucose 11.1 and HEPES 10, pH 7.4 with NaOH. The reference drugs selected for this investigation were obtained from Sigma (CA, USA), Tocris Bioscience (MN, USA) and Cayman Chemical (MI, USA). Drugs were initially formulated in DMSO as a 1000x stock solution. Stock solutions were diluted to the working concentrations in 0.1% DMSO on the day of the experiment.

## Supplementary information

Supplementary information.
