# Supplementary material for: Multiparametric Mechanistic Profiling of Inotropic Drugs in Adult Human Primary Cardiomyocytes
Source: Sci Rep. 2020 May 6;10:7692. doi: 10.1038/s41598-020-64657-2 (PMC7203129; doi:10.1038/s41598-020-64657-2)
Supplement: Supplementary file 1 — Supplementary information. [file 41598_2020_64657_MOESM1_ESM.pdf]

Multiparametric Mechanistic Profiling of Inotropic Drugs in Adult Human Primary Cardiomyocytes

Najah Abi-Gerges[1*], Tim Indersmitten[1], Ky Truong[1], William Nguyen[1], Phachareeya Ratchada[1], Nathalie Nguyen[1,2], Guy Page[1], Paul E Miller[1], Andre Ghetti[1]

[1] AnaBios Corporation, San Diego, CA 92109, USA

Running title: Human primary cardiomyocytes and drug discovery

[*]Correspondence and requests for material should be addressed to:
Dr. Najah Abi-Gerges
3030 Bunker Hill St., Suite 312
San Diego, CA 92109 USA
Tel: +1 858-366-8374
Email: Najah.abigerges@anabios.com

[2] Current address: Arena Pharmaceuticals, San Diego, CA 92121, USA

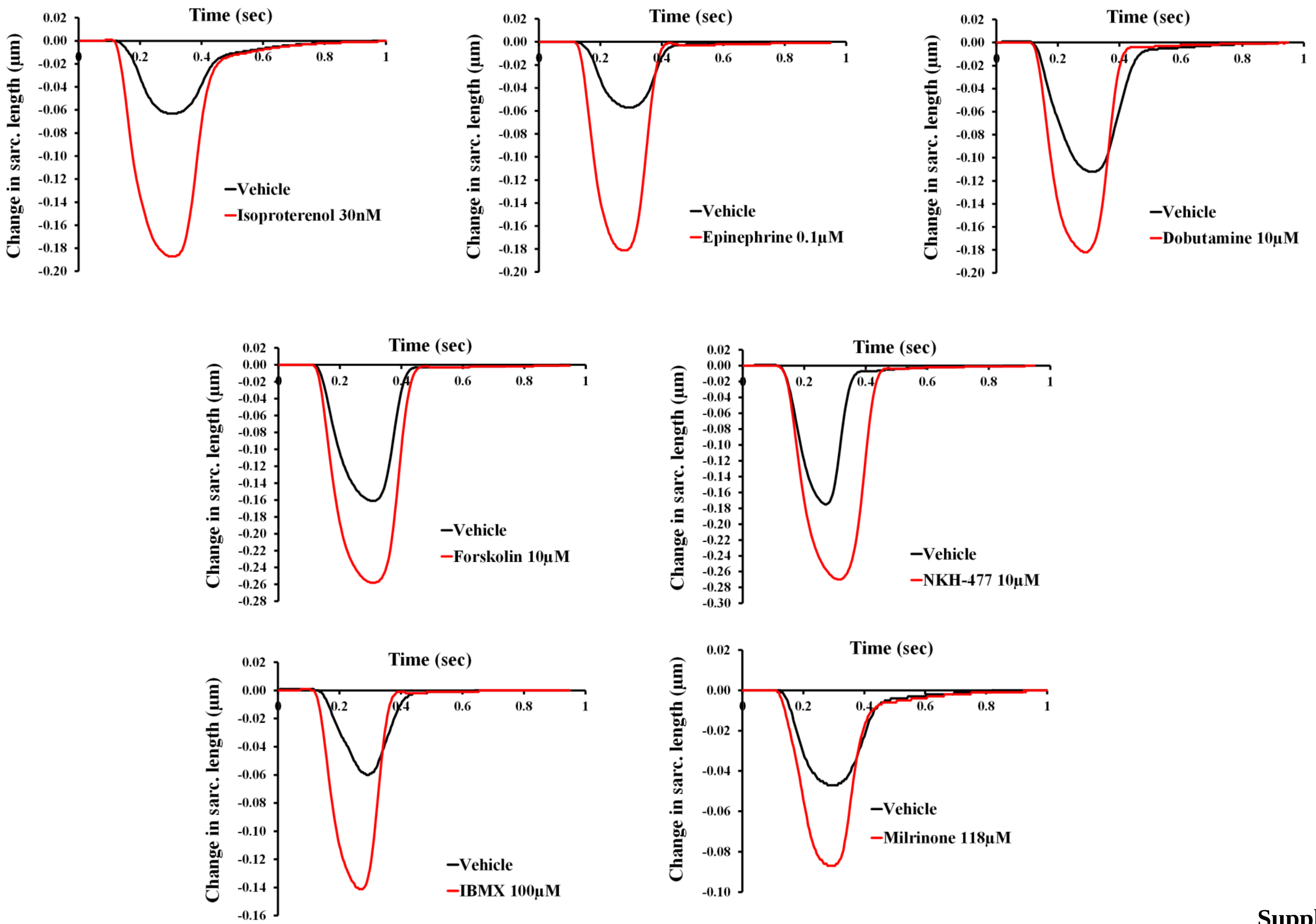

Supplementary Figure 1

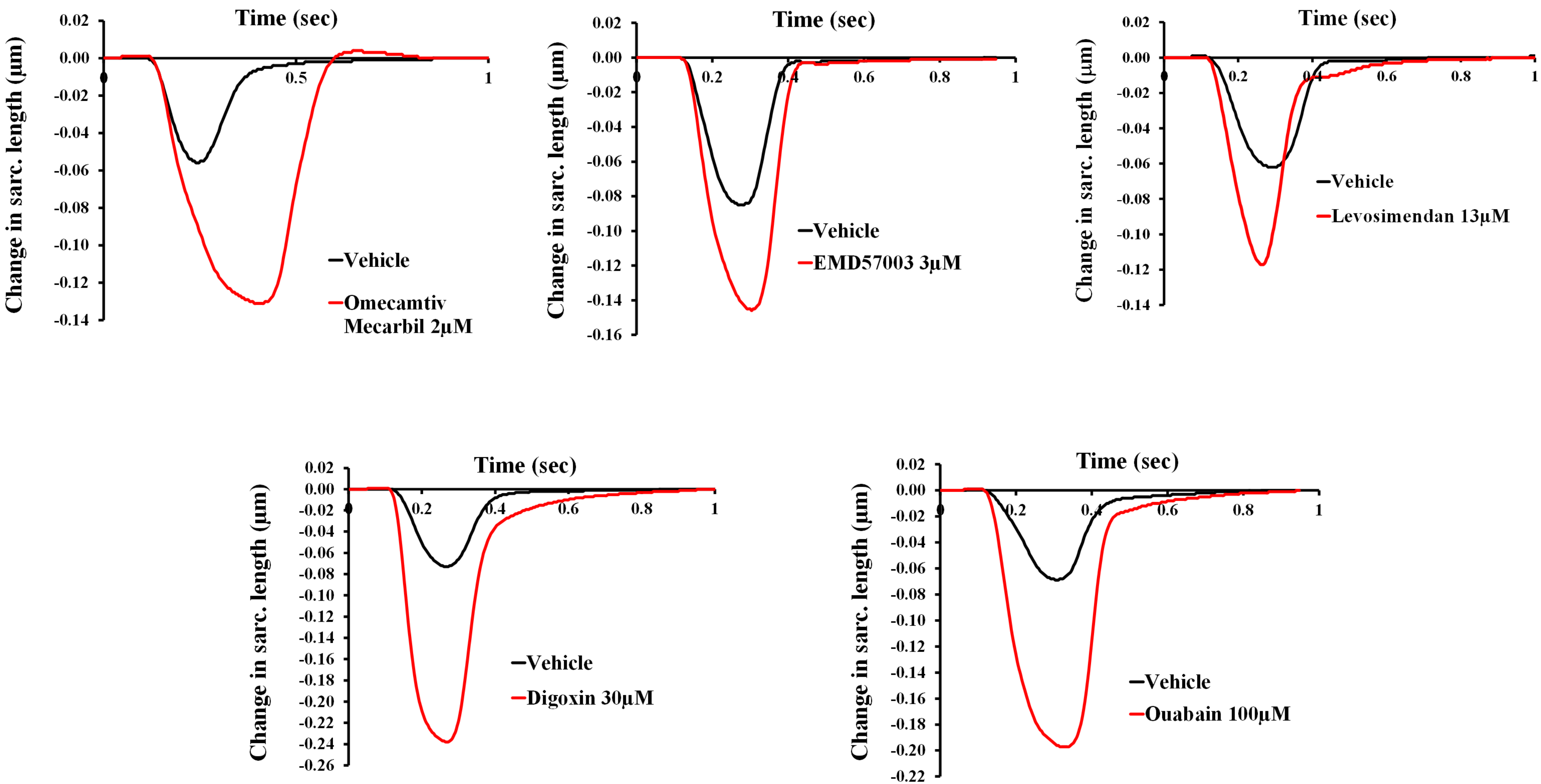

Supplementary Figure 2

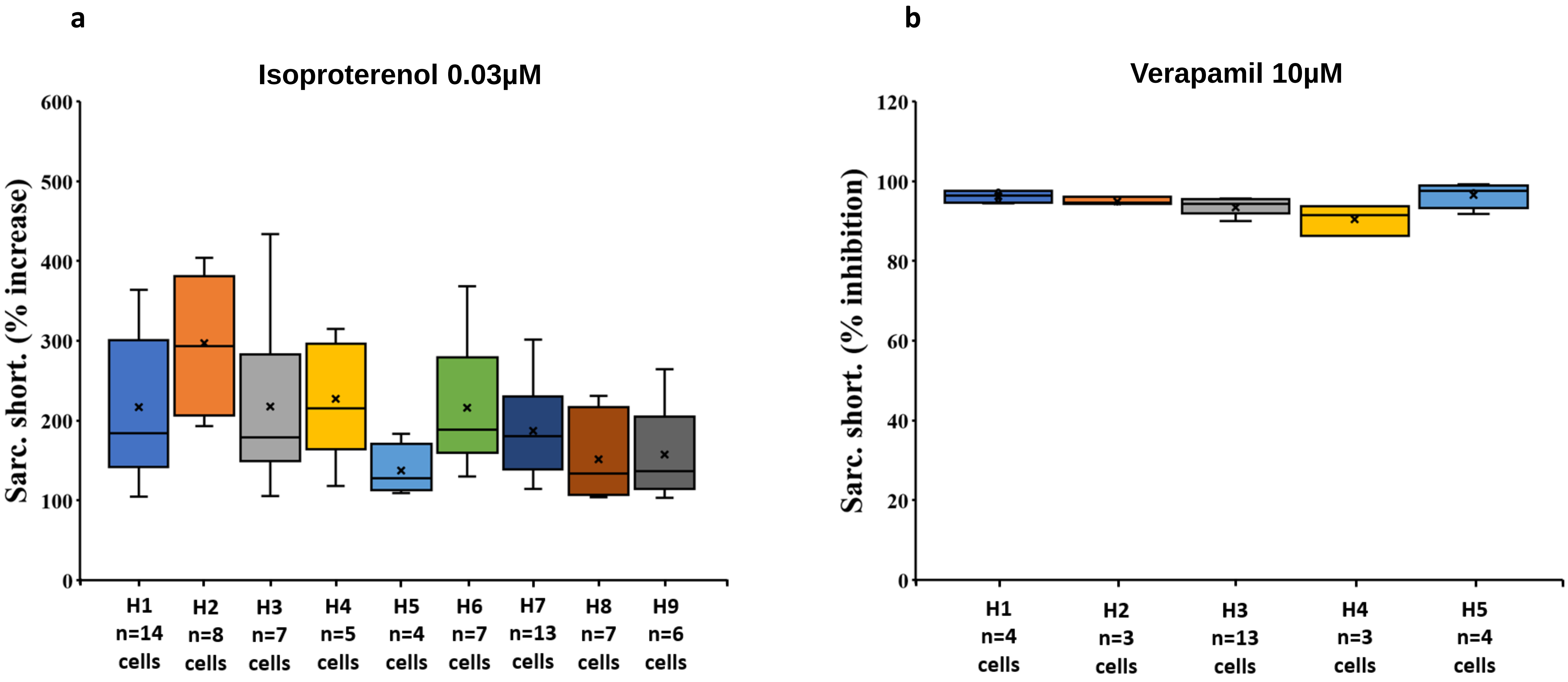

Supplementary Figure 3

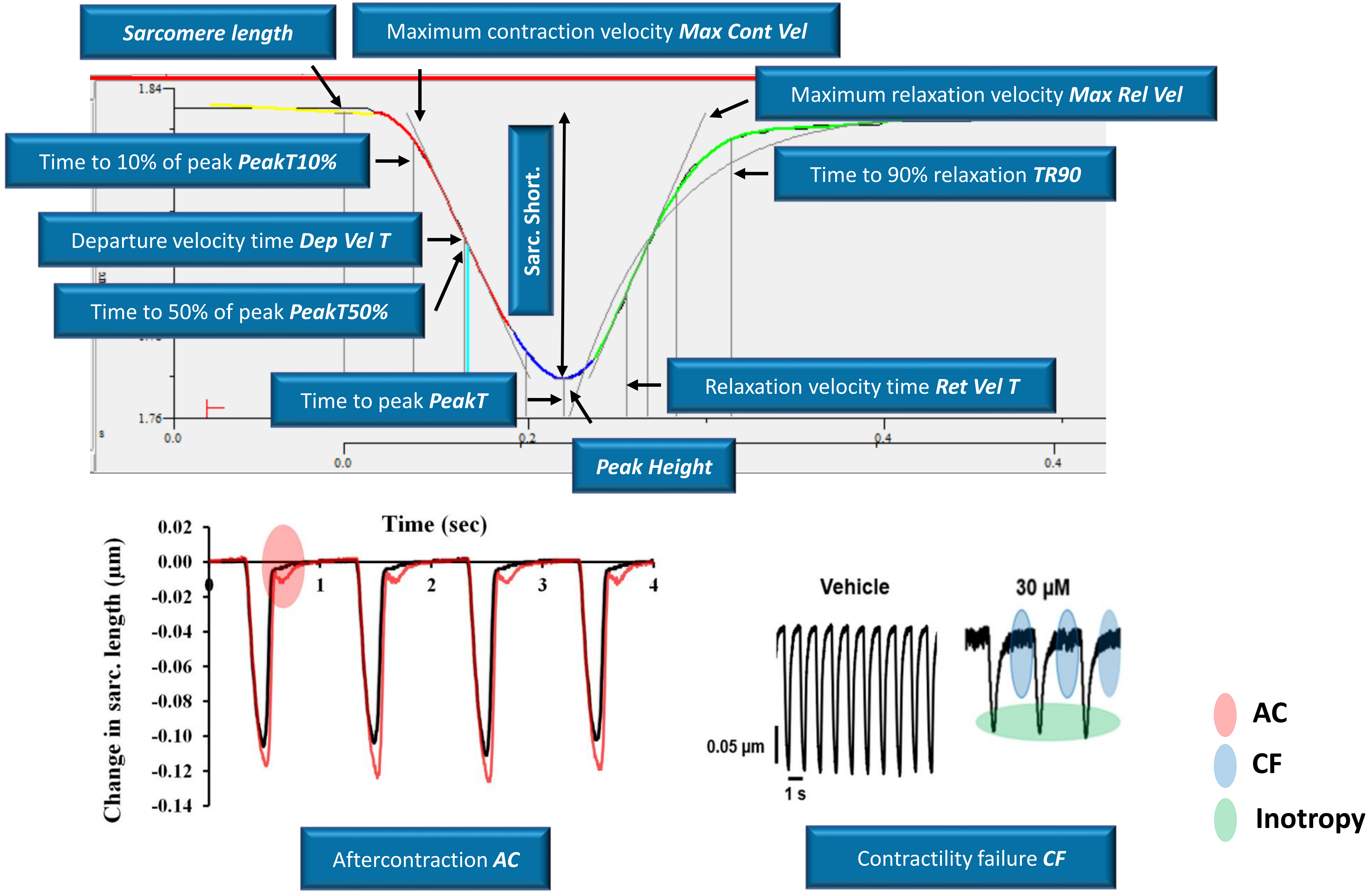

Supplementary Figure 4

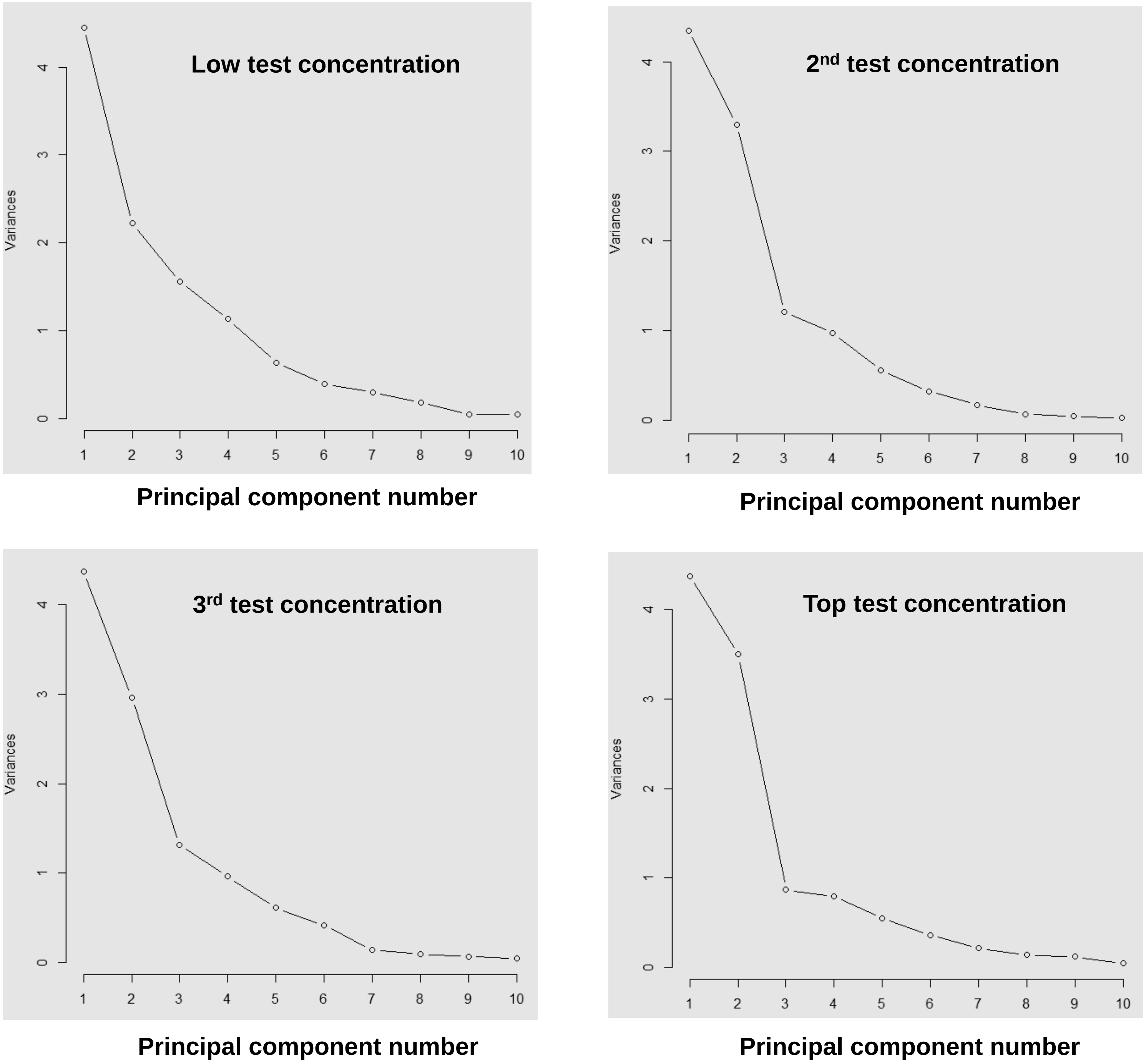

Supplementary Figure 5

Supplementary Table 1 Donor characteristics for 0.03µM Isoproterenol and 10µM Verapamil data

Heart #	Donor identifier	Age	Sex	Ethnicity	BMI	COD	EF (%)	Drug	n
1	190912HHA	46	M	Hispanic	46.1	CVA / ICH	65	Isoproterenol	14
2	190920HHA	40	M	Caucasian	23.8	CNS Tumor	N/A[a]	Isoproterenol	8
3	190923HHA	33	F	Caucasian	18.9	HT / BI	60	Isoproterenol	7
4	190925HHA	53	F	Caucasian	25.6	CVA / ICH	50	Isoproterenol	5
5	190929HHA	37	M	Hispanic	30.5	Anoxia	60	Isoproterenol	4
6	191003HHA	56	F	Caucasian	24.7	Anoxia	70	Isoproterenol	7
7	191010HHA	54	F	Hispanic	60.5	CVA / ICH	63	Isoproterenol	13
8	191021HHA	35	F	Caucasian	20.7	Anoxia	65	Isoproterenol	7
9	191101HHB	44	F	Hispanic	23.9	Anoxia	70	Isoproterenol	6
10	191202HHA	42	M	African American	20.7	CVA / ICH	55	Verapamil	4
11	180523HHA	32	F	Caucasian	23.5	HT	63	Verapamil	3
12	171025HHA[b]	33	M	Caucasian	30.3	CVA / ICH	60	Verapamil	13
13	161204HHA	23	F	Caucasian	19.5	Anoxia	55	Verapamil	3
14	160613HHB	32	F	Caucasian	20.5	Anoxia	50	Verapamil	4

F, Female; M, Male; BMI, Body Mass Index; COD, Cause Of Death; EF, Ejection Fraction; CVA, Cerebrovascular Accident; ICH, Intracranial Haemorrhage; CNS, Central Nervous System; HT, Head Trauma; BI, Blunt Injury; HH, Human Heart; HHA, the 1st heart received on the day; HHB, the 2nd heart received the same day; [a]Organ procurement organization could not transplant the heart and consequently no echocardiography was performed; N/A, Not available; n, Number of cells per donor heart. [b]See Table 1.

Supplementary Table 2: Inotropes tested in adult human primary cardiomyocyte contractility-based model

Drug name	Inotropic effect	Mechanism of action	Concentrations tested (µM)				Cells #	Heart #[a]
Digoxin	Positive	Na+/K+ pump inhibition	0.9	3	9	30	7	2
Ouabain	Positive	Na+/K+ pump inhibition	0.1	1	10	100	5	6
SEA-0400	Positive	Na^+/Ca^{2+} exchanger inhibition	0.1	1	3	10	5	3
Omecamtiv mecarbil	Positive	Myosin activation	0.02	0.2	2	20	5	2
EMD-57003	Positive	Myosin activation	0.1	0.3	1	3	5	6
Levosimendan	Positive	Ca2+ sensitization	0.13	1.3	3.9	13	4	2
Isoproterenol	Positive	β-adrenoceptor activation	0.0003	0.003	0.01	0.03	4	1
Epinephrine	Positive	β-adrenoceptor activation	0.001	0.01	0.03	0.1	4	6
Dobutamine	Positive	β1-adrenoceptor activation	0.01	0.1	1	10	5	5
Milrinone	Positive	PDE3 inhibition	3.5	11.8	35	118	4	1
IBMX	Positive	PDE inhibition	1	10	30	100	5	6
Bay-K 8644	Positive	Ca^{2+} channel activation	0.01	0.03	0.1	0.3	5	5
Forskolin	Positive	Adenylyl cyclase activation	0.001	0.01	0.03	0.1	5	6
NKH-477	Positive	Adenylyl cyclase activation	0.1	1	10	30	6	6
N106	Positive	SERCA activation	0.01	0.1	1	10	5	5
Caffeine	Positive	RyR activation	10	100	1000	10000	5	4, 7
Ryanodine	Negative	RyR inhibition	0.01	0.1	1	10	4	5
Thapsigargin	Negative	SERCA inhibition	0.1	1	10	30	4	2
Flecainide[9]	Negative	Na^+ channel inhibition	0.75	2.26	7.53	22.6	3	8
Mexiletine[9]	Negative	Na^+ channel inhibition	0.25	2.5	25	75	7	9
Diltiazem[9]	Negative	Ca^{2+} channel inhibition	0.13	0.38	1.28	3.84	4	8
Mibefradil[9]	Negative	Ca^{2+} channel inhibition	0.012	0.036	0.12	0.36	6	10
Nifedipine[9]	Negative	Ca^{2+} channel inhibition	0.007	0.023	0.077	0.23	4	10
Nitrendipine[9]	Negative	Ca^{2+} channel inhibition	0.003	0.009	0.03	0.09	4	8
Verapamil	Negative	Ca^{2+} channel inhibition	0.01	0.1	1	10	5	1
$CaCl_2$	Negative/positive	Hypocalcaemia/hypercalcemia	300	1000	3000	10000	5	5

[9]Nguyen N. et al., 2017; #, Number; [a]See Table 1 for Donor Characteristics.
